# When Genes Misfire: ARV1 and the Unseen Battle Against Pediatric Epileptic Encephalopathy

**DOI:** 10.7759/cureus.82297

**Published:** 2025-04-15

**Authors:** Raafat Hammad Seroor Jadah, Jood A Al Aghawani

**Affiliations:** 1 Pediatric Neurology, Bahrain Defence Force Hospital, Riffa, BHR; 2 Medicine and Surgery, Royal College of Surgeons in Ireland - Bahrain, Busaiteen, BHR

**Keywords:** neurological manifestations, pediatric genetics, rare genetic disorder, rare mutation, seizures

## Abstract

The rare ARV1 gene encodes a protein that is crucial for homeostasis and sterol metabolism. It is vital for maintaining membrane integrity and cellular stability. Given the limited epidemiological data, it is evident that ARV1 mutations are rare, showing significant neurological and systemic manifestations, including developmental delays, epilepsy, or cardiomyopathy. We report a case of a six-month-old female presenting with global developmental delay, hypotonia, and poor fine motor milestones. MRI revealed bifrontal subarachnoid spaces and abnormalities in the right parietal lobe. A homozygous pathogenic variant in the ARV1 gene (p.Phe144Argfs*5) was confirmed through whole exome sequencing (WES), thereby diagnosing autosomal recessive developmental and epileptic encephalopathy-38 (DEE38). Through this report, we aim to highlight the importance of early diagnosis in rare genetic disorders and increase awareness among healthcare professionals.

## Introduction

The ARV1 gene is an uncommon ultra-rare gene that encodes a protein that is vital for membrane integrity. It plays a crucial role in lipid homeostasis and the metabolism of sterol. This essentially refers to the synthesis, regulation, and utilization of cholesterol in the body. Cholesterol is a crucial component of cell membranes, a precursor for steroid hormones, vitamin D, and bile acids, making its regulation vital for overall health [1]. The incidence in general populations is not well established due to the scarcity of reported cases and the absence of epidemiological studies. As this mutation has only been recently reported for the first time by Alazami et al. in 2016, each new case reported adds significantly to the literature about this condition [2]. Based on the cases reported so far, patients with ARV1 gene mutation mainly experienced neurological deficits [3,4]. A case reported by Kamate and Basavanagowda described a 14-year-old girl, born to a second-degree consanguineous couple with a normal birth history, who had seizures from the age of five months with clinical manifestations including ataxia, oculomotor apraxia, elevated alpha-fetoprotein (AFP), and a homozygous missense mutation in ARV1 [4]. Global developmental delay, including delay in achieving milestones and cardiomyopathy, is also noted in other studies [3,4].

Based on the literature, a total of 28 cases of ARV1-related disorders have been reported globally up to 2024 [4]. The unique features of ARV1-associated gene mutations include elevated AFP levels and ocular abnormalities, as well as movement disorders in the form of ataxia or dystonia [3]. Whole exome sequencing (WES) has emerged as the most effective diagnostic tool for identifying genetic mutations, given its ability to comprehensively analyze coding regions which harbour 85% of disease-causing mutations, particularly in rare neurogenetic disorders, In addition, WES offers a cost-effective and comprehensive approach to accurately detecting the underlying genetic etiology, which makes it the gold-standard diagnostic tool [5,6]. This case report aims to enhance the understanding of the rare ARV1 gene mutation to increase awareness among physicians and highlight the importance of early intervention in these patients.

## Case presentation

A six-month-old female presented for investigation due to global developmental delay. The patient had no prior history of abnormal movement, seizures, or feeding difficulty. The infant had been born at 37 weeks of gestation, weighing three 3 kilograms with no reported perinatal complications or neonatal intensive care unit (NICU) admission. She had been born via a marriage between closely related individuals and had a healthy four-year-old brother with no family history of epilepsy or special needs. Her vaccination schedule was up to date, and she was on formula feeds and soft solid foods. The infant's mother had first noted poor head support, inability to follow an upward gaze, and generalized floppiness at the age of three months.

**Figure 1:**
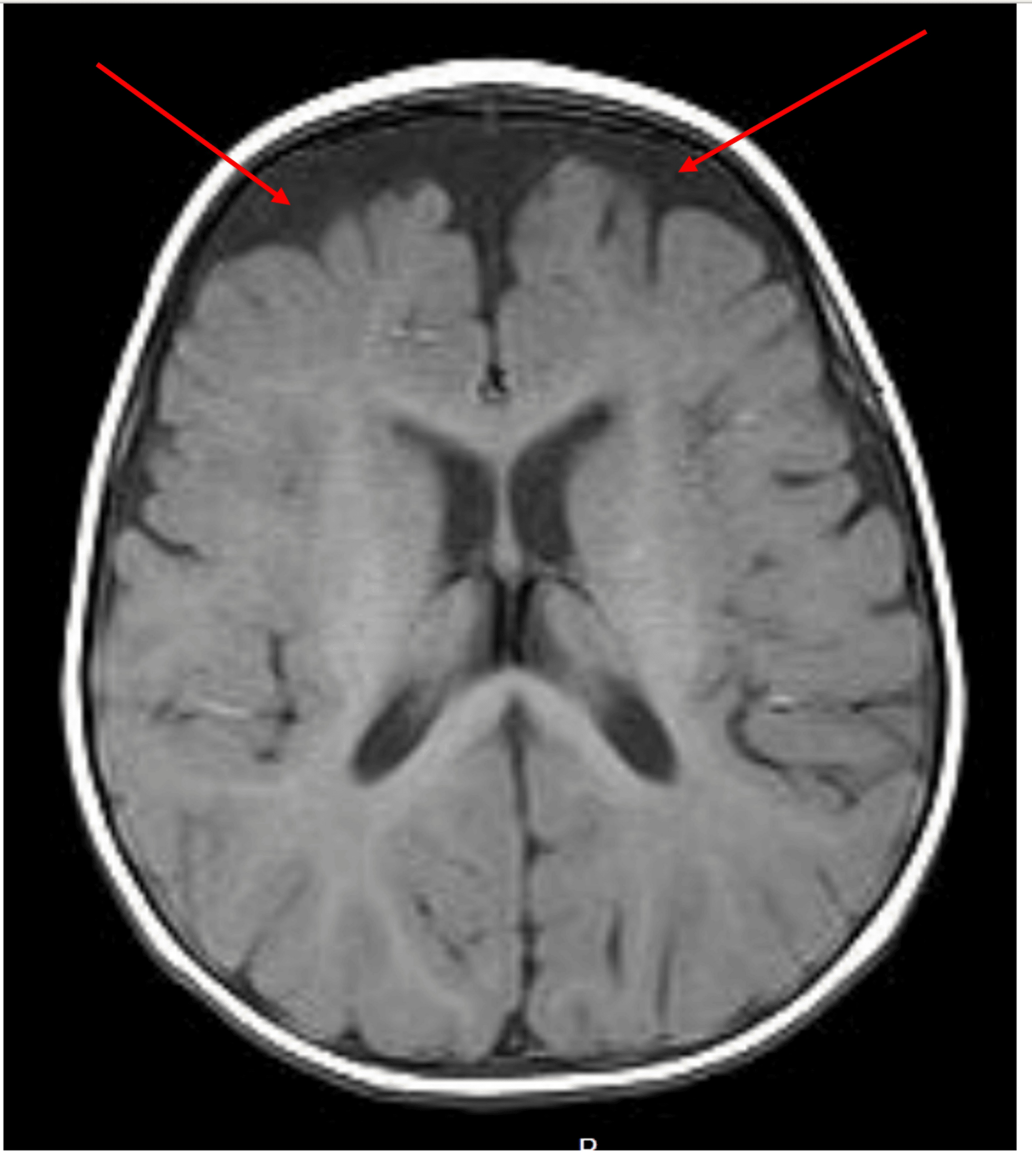
T1 sequence brain MRI - axial view The image shows increased EA-CSF volume in the subarachnoid spaces over the bilateral frontal lobes EA-CSF: extra-axial cerebrospinal fluid; MRI: magnetic resonance imaging

**Figure 2:**
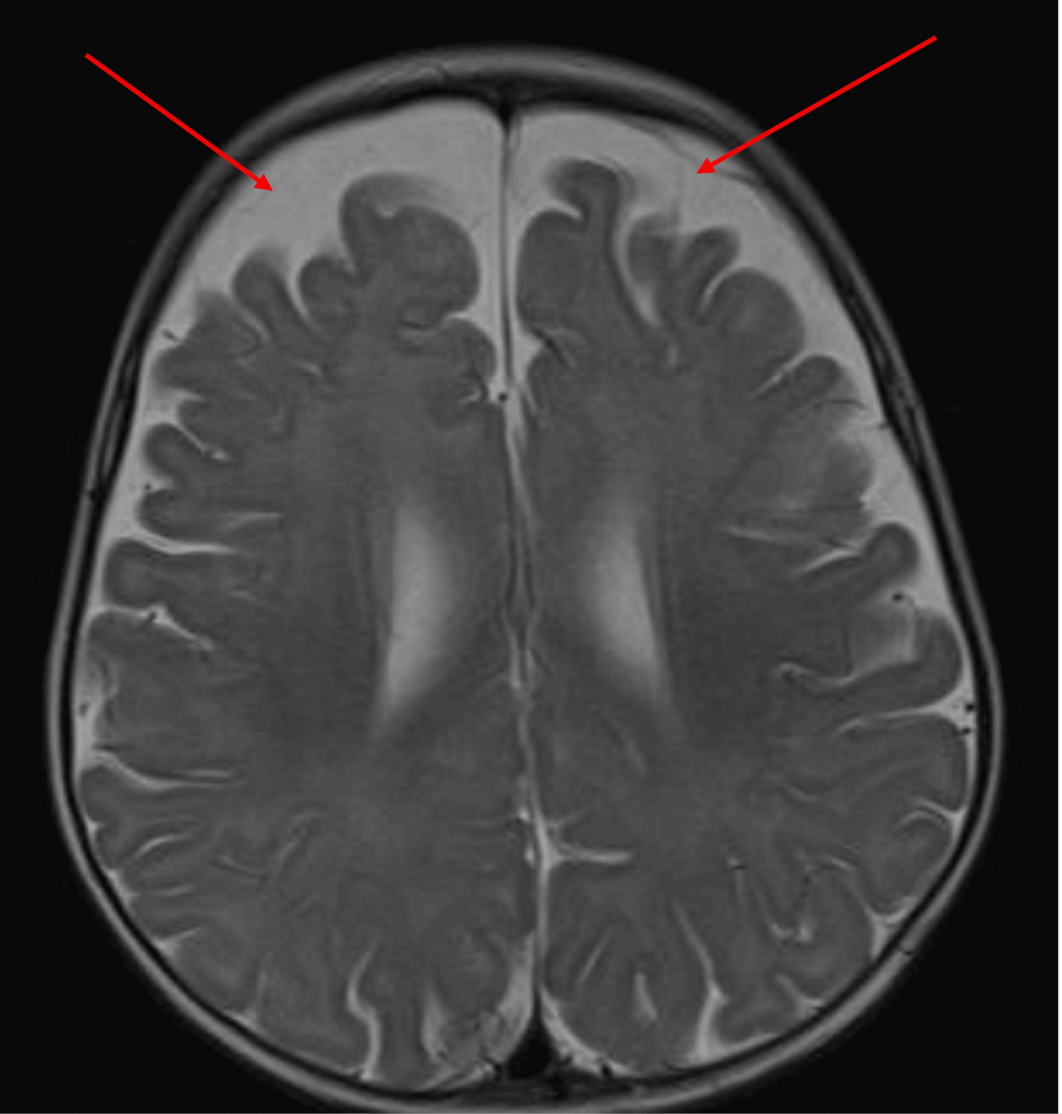
T2 sequence brain MRI - axial view The image shows increased EA-CSF volume in the subarachnoid spaces between the dura and cortical surface over the bilateral frontal lobes EA-CSF: extra-axial cerebrospinal fluid; MRI: magnetic resonance imaging

The initial physical examination showed an active child with an anterior fontanelle at level with subtle dysmorphic features in the form of micrognathia and low-set ears with no neurocutaneous skin lesions. Inability to roll from supine to prone position was noted, as well as failure to fixate and follow upward gaze. She was able to grasp objects briefly and could produce cooing sounds; however, babbling had not been observed yet. She had generalized hypotonia, which is evident in ARV1 mutations, with a normal power of 5/5 in the upper and lower limbs, normal deep tendon reflexes of +2, and no cerebellar signs. The rest of the systemic examination was unremarkable, with no abnormalities in other organ systems. The infant experienced a generalised tonic-clonic seizure affecting all limbs, accompanied by loss of consciousness for four minutes. Drooling and upward gaze deviation were observed. This episode was managed with diazepam and phenytoin, followed by levetiracetam (Keppra) as maintenance therapy, administered with an initial loading dose followed by a maintenance dose. An MRI was performed utilizing both T1 and T2 sequences (Figures 1, 2).

Based on the patient's clinical presentation and abnormal neurodevelopmental milestones and brain MRI, WES was done given its reliability in identifying genetic mutations, which revealed a homozygous pathogenic variant in the ARV1 gene (pp.Phe144Argfs*5), thereby confirming a diagnosis of autosomal recessive developmental and epileptic encephalopathy-38 (DEE38). Tandem mass spectrometry (TMS) excluded metabolic disorders. The patient remained clinically stable without any further seizures while on antiepileptic therapy. Genetic counselling was provided to the family, and the patient was discharged in good condition for follow-up care.

## Discussion

The ARV1 gene encodes a protein involved in lipid homeostasis and regulation of sterol transport within cellular membranes, particularly facilitating transport within the endoplasmic reticulum and plasma membrane. Proper lipid distribution is essential for membrane fluidity and cellular stability; hence, a loss of function in ARV1 disrupts sterol homeostasis, resulting in a build-up of lipids in the endoplasmic reticulum and deficiencies in other membranes, leading to neurological and systemic symptoms [7]. ARV1 deficiency leads to widespread neuronal dysfunction, and the dysregulation in lipids contributes to altered membrane composition, resulting in impaired synaptic activity. The development of epilepsy is thought to be due to the disruption of lipid-dependent signalling pathways, which are vital for maintaining excitatory-inhibitory balance in the brain [8].

Our case report highlights the key features of the ARV1 gene mutation and adds to the growing body of literature associated with the management of challenges associated with these mutations. The clinical presentation in this case aligns with previously reported cases. A case report in 2017 described two siblings with severe developmental delays, generalised hypotonia, and early-onset epilepsy caused by ARV1 mutations [1]. Puusepp et al. have emphasized the role of ARV1 in lipid homeostasis and the resulting neurological dysfunction in affected individuals [3]. Both these findings are consistent with the neurological and developmental deficit seen in our patient. Table 1 below summarizes key findings across ARV1 genetic mutations.

**Table 1:** Key findings from various studies focusing on ARV1 gene mutation Table summarizing the phenotypic and genetic findings in patients with ARV1 mutations, as reported by Alazami et al. [1], Kamate et al. [4], Karabinos et al. (including the case previously described by Segel et al.) [5], and Palmar et al. [6] Genetic variants include homozygous missense mutations (p.Gly189Arg) and homozygous splice site variants, both predicted to impair ARV1 protein function and contribute to the neurodevelopmental phenotype c.565G>A: single-nucleotide variant in the ARV1 gene (guanine replaced by adenine at position 565 in coding DNA sequence); DEE38: developmental and epileptic encephalopathy-38; MRI: magnetic resonance imaging

Study	Patient demographics	Clinical features	MRI findings	ARV1 genetic variant	Inhertiance
Alazmi et al.	3 related children (consanguineous family)	Early infantile seizure; profound intellectual disability and ataxia; one child had visual impairment and one died by age 4	Not reported	Homozygous missense p.Gly189Arg	Autosomal recessive - homozygous in a consanguineous family
Palmer et al.	1 patient (origin not specified)	Severe neurodevelopmental delay; infantile-onset intractable seizures; movement disorder with retinal dystrophy; died at age 1	Not reported	Homozygous splice site variants	Autosomal recessive – biallelic loss of function
Segel et al.	2 brothers (aged 11 and 18 years)	Severe intellectual disability; epilepsy; DEE38; autistic regression in childhood; notably, no significant ophthalmologic abnormalities; milder neurocognitive course compared to patients with splice variants; both developed dilated cardiomyopathy	Not specified	Homozygous missense Gly189Arg	Autosomal recessive
Karabinos et al.	21-year-old male of European descent	Developmental delay and moderate intellectual disability; childhood-onset seizures (DEE38); impaired walking (ataxic gate) and speech; dilated cardiomyopathy in adolescence	MRI in late adolescence showed stable cerebellar atrophy with prominent cerebellar folia; incidental pituitary microadenoma was noted without any other brain structural abnormalities	Homozygous in frame deletion	Autosomal recessive
Kamate et al.	14-year-old (2nd-degree consanguineous marriage, India)	Infantile-onset seizures from 5 months (partially controlled with medication); developmental delay and intellectual disability; onset of ataxia by age 3; oculomotor apraxia and gaze	Cerebellar atrophy on brain MRI	Homozygous missense c.565G>A	Autosomal recessive

All listed cases involve an autosomal recessive inheritance pattern, particularly in consanguineous families with phenotypes ranging from DEE38 with severe neurodevelopmental impairments to milder presentations with ataxia and some missense mutations. These cases collectively expand the spectrum of ARV1-related disorders and highlight vital genotype-phenotype correlations. Distinctive features in our case included early-onset seizures, global developmental delay, as well as dysmorphic features. This report highlights the importance of advanced neuroimaging and genetic testing as they were key factors in the confirmation of the diagnosis. The treatment predominantly focuses on symptom control and supportive management. Further research is essential to explore targeted therapies. As reported by Al Teniji et al., despite optimal seizure control, neurodevelopmental outcomes often remain poor [1]. We observe that genetic counseling and multidisciplinary care, as provided in this case, are essential in supporting affected families and optimizing patient outcomes. This is supported by a recent review by Vasquez and Fine, which stated that "multidisciplinary care in DEEs is paramount" for improving long-term outcomes and described how such an interdisciplinary approach helps maximize developmental skills, mobility, and the overall quality of life [9].

## Conclusions

This case report adds to the expanding corpus of literature on illnesses linked to rare ARV1-related mutations, emphasizing their phenotypic variability and the critical need for comprehensive diagnostic approaches. The recent recognition of associated features such as cardiomyopathy and cerebellar ataxia broadens the understanding of this condition, underscoring the necessity for continued investigations into its underlying mechanisms and therapeutic options. Further studies are warranted to understand the molecular basis of ARV1 deficiency and explore targeted therapies to address both neurological and systemic manifestations. Finally, regarding management, it is evident that the integration of neurorehabilitation with a multidisciplinary care framework may play a pivotal role in enhancing clinical outcomes and improving the quality of life of affected individuals.
